# Evaluation of Health Literacy Levels and Associated Factors Among Patients with Acute Coronary Syndrome and Heart Failure in Qatar

**DOI:** 10.2147/PPA.S385246

**Published:** 2023-01-07

**Authors:** Marwa Elbashir, Maguy Saffouh ElHajj, Daniel Rainkie, Nadir Kheir, Fatima Hamou, Sara Abdulrhim, Ahmed Mahfouz, Sumaya Alyafei, Ahmed Awaisu

**Affiliations:** 1 Department of Clinical Pharmacy and Practice, College of Pharmacy, QU Health, Qatar University, Doha, Qatar; 2 Pharmacy Department, Airport Health Center, Primary Health Care Corporation, Doha, Qatar; 3 Faculty of Pharmaceutical Sciences, University of British Columbia, Vancouver, BC, Canada; 4 College of Pharmacy and Health Sciences, Ajman University, Ajman, United Arab Emirates; 5 Pharmacy Department, Heart Hospital, Hamad Medical Corporation, Doha, Qatar; 6 Pharmacy Department, Aspetar Orthopedic and Sports Medicine Hospital, Doha, Qatar

**Keywords:** health literacy, cardiovascular diseases, acute coronary syndrome, heart failure

## Abstract

**Purpose:**

To determine the prevalence of inadequate health literacy and its associated risk factors among patients with acute coronary syndrome (ACS) and/or heart failure (HF) in Qatar.

**Patients and Methods:**

This cross-sectional observational study was conducted among patients with ACS and/or HF attending the national Heart Hospital in Qatar. Health literacy was assessed using the abbreviated version of the Test of Functional Health Literacy in Adults (S-TOFHLA) and the Three-item Brief Health Literacy Screen (3-item BHLS).

**Results:**

Three hundred patients with ACS and/or HF, majority male (88%) and non-Qatari (94%), participated in the study. The median (IQR) age of the participants was 55 (11) years. The prevalence of inadequate to marginal health literacy ranged between 36% and 54%. There were statistically significant differences in health literacy level between patients based on their marital status (p=0.010), education (p≤0.001), ability to speak any of Arabic, English, Hindi, Urdu, Malayalam, or other languages (p-values ≤0.001 to 0.035), country of origin (p≤0.001), occupation (p≤0.001), and receiving information from a pharmacist (p=0.008), a physiotherapist (p≤0.001), or a nurse (p=0.004).

**Conclusion:**

Inadequate health literacy is common among patients with ACS and/or HF. This study suggests a need for developing strategies to assist healthcare professionals in improving health literacy skills among patients with ACS and HF. A combination of interventions may be needed to improve patients’ understanding of their disease and medications, and ultimately overall health outcomes.

## Introduction

Cardiovascular diseases (CVDs) are recognized as a leading cause of morbidity and mortality worldwide. Of 54 million deaths that occurred globally in 2013, around 17.3 million were attributed to CVDs.^1^ The leading cause of CVD-related deaths was ischemic heart disease (IHD) (8.2 million deaths),^2^ followed by cerebrovascular disease (8 million deaths).^2^ Furthermore, the cost of CVDs is expected to rise from approximately $863 billion in 2010 to $1044 billion in 2030 globally.^3^ In Qatar, a country with a population of less than 3 million, 16,750 patients were admitted with acute coronary syndromes (ACS) between 1991 and 2010.^4^ Moreover, CVDs were the leading causes of mortality in Qatar, accounting for 17.1 deaths per 100,000 population in 2010.^5^ Although there are existing registries such as Gulf CARE (Gulf Acute Heart Failure Registry), published data on the epidemiology of heart failure (HF) in Qatar and other Gulf Cooperation Council (GCC) countries are scarce.

Literacy in general and health literacy in particular are of significant importance in ensuring patients’ engagement and self-management in chronic diseases. Although high literacy does not always translate to high health literacy and patients with high literacy level may have low to moderate health literacy, yet it is important to gauge both aspects from health management imperatives. In Qatar with a population of 2,891,000 (November 2022), literacy rate is very high among the general population and has been reported to be 93.5% among individuals ≥15 years and 70.5% among elderly (≥65 years). However, health literacy level is not directly reported in medical records of patients with ACS/HF or other diseases.

CVDs, including, but not restricted to ACS and HF, are complex conditions that require patients’ high-level of involvement^6^,^7^ as well as knowledge and understanding to manage their disease conditions.^8^ Therefore, the promotion of self-management, which is “the individual’s ability to manage symptoms, treatment, physical and psychosocial consequences, and lifestyle changes inherent in living with a chronic condition”, is essential in this patient population.^9^ Inadequate health literacy hinders many patients with ACS and HF from engaging in effective self-care management of their conditions.^10^,^11^ Patients with limited health literacy tend to use a passive communication style with their healthcare providers, do not participate in decision-making, and lack the ability to self-manage their diseases and medications.^12^ According to a pooled analysis of 85 studies, the prevalence of low health literacy and marginal health literacy was 26% and 20%, respectively.^13^ According to other studies, low health literacy ranged from 17.5% to 97% among patients with HF^14^ and from 34% to 44% among patients with ACS.^15^,^16^ In the last 20 to 30 years, studies have shown that low or inadequate health literacy, compared to adequate health literacy, has been associated with poorer knowledge, health outcomes, and comprehension of healthcare services.^17^ For instance, low health literacy was found to be associated with lower medication adherence,^18^ increased incidence of hospitalization,^19^,^20^ and higher risk of mortality^21^ in patients with HF. Similarly, patients with ACS and limited health literacy were found to have lower medication adherence^22^ and higher hospital readmission rates.^23^,^24^

Therefore, assessment of health literacy in patients with HF and ACS is crucial, in order to ensure optimal therapeutic outcomes. In general, health literacy assessment instruments are broadly categorized into generic and disease-specific tools. Generic instruments are for general use to assess health literacy across different patient populations.^25^ Some of the most common types of health literacy assessment instruments include word-recognition tests and tests of functional health literacy. Word-recognition tests measure an individual’s ability to recognize and pronounce words in a list and are considered useful predictors of general reading ability. One of the most commonly used word-recognition tests is the Rapid Estimate of Adult Literacy in Medicine (REALM), which is a list of 66 medical terms that can be completed in 3 to 5 minutes.^26^ Tests of functional health literacy include word-recognition, reading comprehension, numeracy skills, and application to real-life situations. Even though these measures are used to assess health literacy among different populations, their applicability and appropriateness among patients with CVDs are not widely studied. A systematic review published in 2018 identified the health literacy assessment instruments available and used in coronary artery disease (CAD).^27^ Another systematic review identified the available evidence pertaining to the instruments that have been used to measure health literacy in patients with CVDs.^28^ In this review, 10 health literacy assessment instruments used among patients with CVD in the included studies (n = 42) were generic, only one instrument, the High Blood Pressure-Health Literacy Scale (HBP-HLS), was disease-specific (n = 1).^28^

Adequate health literacy is essential for adherence and better health outcomes among patients with CVDs. The issue of health literacy has been widely studied in developed countries, with very few studies conducted in the Arab world. Therefore, evidence on the level of health literacy among patients with CVDs is limited in Qatar and the larger Middle East region. The high prevalence and burden of CVDs in the region, especially ACS and HF, merits investigations to assess health literacy in an effort to determine effective strategies to improve communication and health outcomes in this population. Therefore, this study aimed to: (1) assess the level of health literacy among patients with ACS and/or HF using adapted validated health literacy assessment instruments; (2) determine the prevalence of limited health literacy in this population and; (3) determine the patient characteristics that are associated with the level of health literacy (i.e. to compare the characteristics of patients with limited health literacy versus those with adequate health literacy).

## Patients and Methods

### Study Design and Setting

This was a cross-sectional descriptive study in which patients’ health literacy levels were measured using two health literacy assessment instruments (the abbreviated version of the Test of Functional Health Literacy in Adults [S-TOFHLA] and the Three-item Brief Health Literacy Screen [3-item BHLS]). The data were collected between 1 April 2019 and 30 August 2019 at the Heart Hospital, a member of Hamad Medical Corporation (HMC) in Qatar. The Heart Hospital is a specialist tertiary hospital that provides care in cardiology and cardiothoracic surgery for adult population of Qatar.^29^

### Study Population and Eligibility Criteria

The target population for the study was adult patients diagnosed with ACS, HF, or both disease conditions and receiving care at the Heart Hospital in Qatar. Patients were enrolled in the study if they were 18 years or older, diagnosed with ACS, HF, or both, and were outpatients receiving treatment at the Heart Hospital. Both newly diagnosed patients and those with pre-existing history of ACS and/or HF were included in the study. Patients were excluded from the study if they met any of the following criteria: documented sight impairment, hearing impairment, cognitive difficulty, or patients who do not speak any of the study languages.

### Sample Size and Sampling Technique

The sample size calculation followed the cross-sectional study design for qualitative variables^30^ using a level of confidence of 95%, type-1 error of 5%, and prevalence of limited health literacy of 19%.^31^,^32^ The minimum effective sample size required was calculated to be 237 according to the above assumptions. To account for missing data, a 30% increase in the sample size was targeted. Therefore, a convenient sample of 300 patients with ACS and/or HF was recruited. Eligible participants were identified through an electronic medical records database, CERNER. Patients were approached and recruited from the outpatient department while waiting to be seen by their healthcare providers in follow-up cardiology clinics. Patients who provided an informed consent to participate in the study and fulfilled the eligibility criteria were included in this study.

### Outcome Measures

The primary outcome measure was patient’s health literacy level. Health literacy was assessed using the Abbreviated version of the Test of Functional Health Literacy in Adults (S-TOFHLA) and the Three-item Brief Health Literacy Screen (3-item BHLS). The two different health literacy assessment instruments were utilized concurrently for triangulation purposes.

### Study Instrument

The data collection tool for this research project consisted of three sections: baseline demographic and clinical characteristics section, the S-TOFHLA section (36 items), and the 3-item BHLS section (three items). The S-TOFHLA and 3-item BHLS were selected, because they are commonly used validated and reliable instruments for the assessment of health literacy.^33^,^34^ Furthermore, S-TOFHLA and 3-item BHLS, which were originally developed in English, were translated and validated into Arabic.^35^ The most commonly used functional health literacy assessment instrument is TOFHLA.^28^,^36^ This measure takes a relatively long time (22 minutes) to complete. The abbreviated version of TOFHLA, S-TOFHLA, takes about 12 minutes to complete and its results were well correlated with the original TOFHLA.^33^ The S-TOFHLA comprises of 36-item reading comprehension and 4-item numeracy. The S-TOFHLA reading comprehension score is from 0–36. Scores of 0–16 and 17–22, respectively, identify patients as inadequate and marginal health literacy, while scores ≥23 identify patients as adequate health literacy. The BHLS score ranges from 0–12 and categorized as 0–6 (inadequate), 7–9 (marginal), and 10–12 (adequate) functional health literacy. Health literacy level is categorized differently according to the assessment tool used (Table 1). Below is a description of the health literacy scoring method that was used for this study:^33^,^34^

- Adequate health literacy: Patients who are able to read, understand, and interpret most health texts.
- Marginal or inadequate health literacy: Patients who have difficulty understanding and/or interpreting most health materials. As a result, they would not be able to follow directions for their health care (e.g. take their medications incorrectly, fail to follow prescribed diets, etc).

**Table 1:** Health Literacy Assessment Tools’ Scoring System*

Score	Level	Functional Health Literacy Description (if any)
S-TOFHLA scoring system*		
0–16	Inadequate functional health literacy	Unable to read and interpret health texts
17–22	Marginal functional health literacy	Has difficulty reading and interpreting health texts
23–36	Adequate functional health literacy	Can read and interpret most health texts
3-item BHLS scoring system*		
0–6	Inadequate functional health literacy	
7–9	Marginal functional health literacy	
10–12	Adequate functional health literacy	

**Note**: *Standard scoring based on the tool’s developers.

**Abbreviations**: S-TOFHLA, short version of the Test of Functional Health Literacy in Adults; BHLS, Brief Health Literacy Screen.

### Data Collection Method

Eligible patients were identified through CERNER. The data collection process lasted for 10 to 15 minutes per patient on average. First, demographic and clinical data were obtained from the CERNER and verified by asking the patient. Then, an interviewer administered the 3-item BHLS. Finally, the S-TOFHLA was administered face-to-face, where the interviewer presented the tool from a scripted introduction. Once introduced, the patient was given the reading comprehension passages and numeric calculations to complete.

### Data Analysis

The collected data were analyzed using IBM Statistical Package for Social Sciences (SPSS^®^) software for Windows version 23.0 (IBM Corp, Armonk, New York, USA). Patients’ demographic information, clinical data, and health literacy scores were presented descriptively as median (IQR) for continuous variables and as counts with percentages for categorical variables. The Mann–Whitney U and the Kruskal–Wallis tests were applied to compare statistical differences in health literacy scores between groups. The association between different variables and health literacy scores were tested using Spearman rho correlation test for continuous or ordinal variables and Pearson’s Chi-square or Fisher’s Exact tests for categorical variables. For all statistical tests, a two-sided P-value <0.05 was considered statistically significant. Cohen’s Kappa test was used to determine the level of agreement between S-TOFHLA and 3-item BHLS.

### Ethical Considerations

Ethics approval was obtained from both the Medical Research Center of the HMC [approval reference number: MRC-02-17-087] and the Institutional Review Board at Qatar University [approval reference number: QU-IRB 955-E/18]. Informed consent was obtained from all participants. Participants were informed that their participation was voluntary and their information will be kept strictly confidential. All the procedures performed in this study were in accordance with good clinical practice, the Declaration of Helsinki, and other comparable ethical standards.

## Results

### Demographic Characteristics

Three hundred patients were enrolled in this study from April to August 2019. The demographic characteristics of the study participants are presented in Table 2. The median (IQR) age of the sample was 55 (11) years, 88% were male, and 94% were non-Qatari. A large proportion of the participants (48%) had completed university education, while about 40% had a maximum of high school education or less. The majority (89%) of the participants can read and write in English, 54% can read and write in Arabic, 41% can read and write in Hindi, and 34% can read and write in Urdu. Furthermore, most participants had received health information within the past 6 months from a physician (93%), a pharmacist (78%), or a nurse (67%).

**Table 2:** Demographic and Clinical Characteristics of the Study Participants (n=300)

Variable	n (%)	Median (IQR)
**Demographic characteristics**		
Age		
<60 years	226 (75.3)	
≥60 years	74 (24.7)	
Gender		
Male	265 (88.3)	
Female	35 (11.7)	
Marital status		
Single	27 (9.0)	
Married	272 (90.7)	
Divorced/widowed/separated	1 (0.3)	
Nationality		
Qatari	17 (5.7)	
Non-Qatari	283 (94.3)	
Country of origin		
India	78 (26.0)	
Egypt	46 (15.3)	
Bangladesh	31 (10.3)	
Others	26 (8.7)	
Pakistan	25 (8.3)	
Qatar	17 (5.7)	
Sudan	14 (4.7)	
Philippines	12 (4.0)	
Syria	12 (4.0)	
Sri Lanka	11 (3.7)	
Jordan	9 (3.0)	
Palestine	8 (2.7)	
Nepal	5 (1.7)	
Lebanon	4 (1.3)	
Iran	2 (0.7)	
Education level		
None	1 (0.3)	
Primary school	7 (2.3)	
Middle school	27 (9.0)	
High school	84 (28.0)	
College/diploma	21 (7.0)	
University	144 (48.0)	
Post-graduate	16 (5.3)	
Languages spoken*		
English	266 (88.7)	
Arabic	161 (53.7)	
Hindi	122 (40.7)	
Urdu	103 (34.3)	
Malayalam	39 (13.0)	
Tagalog	14 (4.7)	
Tamil	3 (1.0)	
Other	60 (20.0)	
Occupation		
Unemployed	21 (7.0)	
Management	27 (9.0)	
Finance/accounting	20 (6.7)	
Medical/healthcare	12 (4.0)	
Driving	20 (6.7)	
Retail salesperson	13 (4.3)	
Retired	17 (5.7)	
Administration	24 (8.0)	
Engineering	29 (9.7)	
Teaching	6 (2.0)	
Labor	39 (13.0)	
Cashier	2 (0.7)	
Secretary	2 (0.7)	
Others	68 (22.7)	
Health information source*		
Physician	280 (93.3)	
Pharmacist	234 (78.0)	
Physiotherapist	29 (9.7)	
Nurse	200 (66.7)	
Dietician	16 (5.3)	
Person in charge of medications (at home)		
Self	298 (99.3)	
Spouse/partner	2 (0.7)	
**Clinical characteristics**		
Diagnosis		
HF only	32 (10.7)	
ACS only	237 (79.0)	
HF and ACS	31 (10.3)	
NYHA classification^†^		
I	13 (21.0)	
II	31 (50.0)	
III	14 (22.6)	
IV	4 (6.5)	
ACS type^†^		
STEMI	90 (39.6)	
NSTEMI	108 (47.6)	
UA	29 (12.8)	
HF duration (years)		2.0 (1.0)
ACS duration (years)		2.4 (4.0)
Comorbidities*		
Diabetes	145 (48.3)	
Hypertension	199 (66.3)	
Dyslipidemia	123 (41.0)	
Renal dysfunction	18 (6.0)	
Liver dysfunction	2 (0.7)	
Atrial Fibrillation	18 (6.0)	
Other	78 (26.0)	
Number of comorbidities		3.0 (2.0)
Chronic medications*		
Beta-blocker	265 (88.3)	
Antiplatelet	281 (93.7)	
Statin	274 (91.3)	
ACEI/ARB	231 (77.0)	
CCB	69 (23.0)	
Diuretic	85 (28.3)	
Other	220 (73.3)	
Number of oral medications		6.0 (3.0)
Smoking status		
Never	169 (56.3)	
Former	73 (24.3)	
Current	58 (19.3)	
Weight (kg)		80.0 (19.4)
Height (cm)		169.0 (10.0)
BMI (kg/m^2^)		28.0 (6.6)
SBP (mmHg)		128.0 (27.0)
DBP (mmHg)		77.5 (13.0)
Heart rate (bpm)		71.0 (16.0)
Total cholesterol (mmol/L)		3.5 (1.5)
LDL (mmol/L)		1.9 (1.2)
HDL (mmol/L)		1.0 (0.4)
TG (mmol/L)		1.4 (1.0)
HbA1c (%)		6.1 (1.9)

**Notes**: *Items are not mutually exclusive (ie multiple options response). ^†^Some missing values.

**Abbreviations**: ACEI, Angiotensin-Converting Enzyme Inhibitors; ACS, Acute Coronary Syndrome; ARB, Angiotensin Receptor Blockers; BMI, Body Mass Index; CCB, Calcium Channel Blockers; DBP, Diastolic Blood Pressure; HbA1c, Glycated Hemoglobin A1c; HDL, High-Density Lipoproteins; HF, Heart Failure; LDL, Low-Density Lipoproteins; NSTEMI, Non-St-Elevation Myocardial Infarction; NYHA, New York Heart Association; SBP, Systolic Blood Pressure; STEMI, ST-Elevation Myocardial Infarction; TG, Triglycerides; UA, Unstable Angina.

### Clinical Characteristics

The clinical characteristics of the study population are presented in Table 2. The majority (89%) of participants had ACS, while 21% had HF. The most commonly reported chronic comorbidities included hypertension (66%), diabetes (48%), and dyslipidemia (41%). The median (IQR) number of comorbidities was 3 (2) diseases. The most commonly reported chronic oral medications used by the participants were antiplatelets (94%), statins (91%), beta-blockers (88%), and angiotensin converting enzyme inhibitor (ACEI) or angiotensin receptor blocker (ARB) (77%) with a median (IQR) number of medications of 6 (3) . The median (IQR) BMI of the study participants was 28 (6.6) kg/m^2^, which is considered as overweight.

### Health Literacy Characteristics

The health literacy characteristics of the study participants are presented in Table 3. Among the participants, 36% had inadequate or marginal health literacy according to S-TOFHLA, while over half (54%) had inadequate or marginal health literacy according to 3-item BHLS. Cohen’s Kappa test indicated a significantly moderate agreement between S-TOFHLA and 3-item BHLS scoring (k=0.46, p≤0.001) (Table 4).

**Table 3:** Health Literacy Characteristics of the Study Participants (n=300)

Variable	n (%)	Median (IQR)
S-TOFHLA score*		31.0 (16.0)
S-TOFHLA category		
Adequate (23–36)	192 (64.0)	
Inadequate or marginal (0–22)	108 (36.0)	
BHLS score^†^		9.0 (4.0)
BHLS category		
Adequate (10–12)	139 (46.3)	
Inadequate or marginal (0–9)	161 (53.7)	

**Notes**: *S-TOFHLA scores range from 0 to 36. ^†^BHLS scores range from 0 to 12.

**Abbreviations**: S-TOFHLA, short version of the Test of Functional Health Literacy in Adults; BHLS, Brief Health Literacy Screen.

**Table 4:** Health Literacy Assessment Agreement Between S-TOFHLA and 3-Item BHLS (n=300)

Health Literacy Test		3-Item BHLS Categories		
		Adequate	Marginal	Inadequate
		n (%)		
**S-TOFHLA categories**	Adequate	132 (95.0)	52 (49.5)	8 (14.3)
	Marginal	4 (2.9)	39 (37.1)	17 (30.4)
	Inadequate	3 (2.2)	14 (13.3)	31 (55.4)

**Abbreviations**: S-TOFHLA, short version of the Test of Functional Health Literacy in Adults; BHLS, Brief Health Literacy Screen.

The patient’s characteristics associated with adequacy of health literacy were determined. The demographic characteristics of patients with adequate versus inadequate or marginal health literacy based on S-TOFHLA are presented in Table 5. Seventy-eight percent of patients with adequate health literacy had either undergraduate or postgraduate university education as compared to less than 10% of patients with inadequate or marginal health literacy (p≤0.001). In addition, 63% of patients with adequate health literacy can read and write in Arabic as compared to 37% of patients with inadequate or marginal health literacy (p≤0.001). Similar results were obtained between the demographic characteristics of adequate versus inadequate/marginal health literacy patients based on BHLS tool. About 91% of patients with adequate health literacy had undergraduate or postgraduate university education as compared to 21% of patients with inadequate or marginal health literacy (p≤0.001). Similarly, 63% of patients with adequate health literacy were literate in Arabic as compared to 46% of patients with inadequate or marginal health literacy (p=0.004).

**Table 5:** Demographic Characteristics Based on S-TOFHLA Category

Variable	Adequate (n=192)	Inadequate or Marginal (n=108)	P-value^†^
	n (%)		
Age			
<60 years	149 (77.6)	77 (71.3)	0.224
≥60 years	43 (22.4)	31 (28.7)	
Gender			
Male	170 (88.5)	95 (88.0)	0.881
Female	22 (11.5)	13 (12.0)	
Marital status			
Single	12 (6.3)	15 (13.9)	0.066
Married	179 (93.2)	93 (86.1)	
Divorced/widowed/separated	1 (0.5)	0 (0.0)	
Nationality			
Qatari	14 (7.3)	3 (2.8)	0.124^‡^
Non-Qatari	178 (92.7)	105 (97.2)	
Education level			
None	0 (0.0)	1 (0.9)	≤0.001
Primary school	0 (0.0)	7 (6.5)	
Middle school	5 (2.6)	22 (20.4)	
High school	25 (13.0)	59 (54.6)	
College/diploma	12 (6.3)	9 (8.3)	
University	136 (70.8)	8 (7.4)	
Post-graduate	14 (7.3)	2 (1.9)	
Languages spoken*			
Arabic	121 (63.0)	40 (37.0)	≤0.001
English	171 (89.1)	95 (88.0)	0.773
Hindi	55 (28.6)	67 (62.0)	≤0.001
Urdu	45 (23.4)	58 (53.7)	≤0.001
Tamil	2 (1.0)	1 (0.9)	1.000^‡^
Tagalog	8 (4.2)	6 (5.6)	0.584
Malayalam	21 (10.9)	18 (16.7)	0.157
Other	26 (13.5)	34 (31.5)	≤0.001
Occupation			
Unemployed	10 (5.2)	11 (10.2)	≤0.001
Management	25 (13.0)	2 (1.9)	
Finance/accounting	19 (9.9)	1 (0.9)	
Medical/healthcare	12 (6.3)	0 (0.0)	
Driving	0 (0.0)	20 (18.5)	
Retail salesperson	4 (2.1)	9 (8.3)	
Retired	13 (6.8)	4 (3.7)	
Administration	20 (10.4)	4 (3.7)	
Engineering	27 (14.1)	2 (1.9)	
Teaching	6 (3.1)	0 (0.0)	
Labor	6 (3.1)	33 (30.6)	
Cashier	1 (0.5)	1 (0.9)	
Secretary	1 (0.5)	1 (0.9)	
Others	48 (25.0)	20 (18.5)	
Health information source*			
Physician	177 (92.2)	103 (95.4)	0.289
Pharmacist	156 (81.3)	78 (72.2)	0.070
Physiotherapist	27 (14.1)	2 (1.9)	≤0.001^‡^
Nurse	136 (70.8)	64 (59.3)	0.041
Dietician	11 (5.7)	5 (4.6)	0.684
Person in charge of medications (at home)			
Self	191 (99.5)	107 (99.1)	1.000^‡^
Spouse/partner	1 (0.5)	1 (0.9)	

**Notes**: *Items are not mutually exclusive. ^†^P-values were calculated using Chi-square test. ^‡^P-values were calculated using Fisher’s Exact test.

**Abbreviation**: S-TOFHLA, short version of the Test of Functional Health Literacy in Adults.

Table 6 presents S-TOFHLA scores across different demographic characteristics, clinical characteristics, and health literacy levels. There was a statistically significant difference in health literacy based on marital status, where the median (IQR) S-TOFHLA score was 19 (16) among single patients compared to 31 (15) among married patients (p=0.010). Similarly, there was a statistically significant difference in health literacy score based on education level, where the median (IQR) score for patients with high school education or less ranged from 16 (6) to 19 (9) as compared to 34 (4) to 35 (3) for patients with undergraduate or postgraduate university education, respectively (p≤0.001). Moreover, the median (IQR) S-TOFHLA scores differed significantly according to whether or not the patient speaks Arabic, English, Hindi, Urdu, Malayalam, or other languages (p-values range from ≤0.001 to 0.035). The S-TOFHLA scores also differed significantly based on country of origin (p≤0.001), occupation (p≤0.001), and whether or not the patient received health information within the past 6 months from a pharmacist (p=0.008), physiotherapist (p≤0.001) or nurse (p=0.004).

**Table 6:** Differences in S-TOFHLA Scores Across Different Demographic Characteristics, Clinical Characteristics, and Health Literacy

Variable	n (%)	Median S-TOFHLA Score (IQR)*	P-value
Age			
<60 years	226 (75.3)	31.0 (15.0)	0.377^‡^
≥60 years	74 (24.7)	28.5 (17.0)	
Gender			
Male	265 (88.3)	31.0 (16.0)	0.682^‡^
Female	35 (11.7)	31.0 (19.0)	
Marital status			
Single	27 (9.0)	19.0 (16.0)	0.010^§^
Married	272 (90.7)	31.0 (15.0)	
Nationality			
Qatari	17 (5.7)	30.0 (8.0)	0.936^‡^
Non-Qatari	283 (94.3)	31.0 (16.0)	
Country of origin			
Qatar	17 (5.7)	30.0 (8.0)	≤0.001^§^
Egypt	46 (15.3)	34.0 (5.0)	
Palestine	8 (2.7)	33.5 (12.0)	
Lebanon	4 (1.3)	34.0 (4.0)	
Syria	12 (4.0)	33.0 (14.0)	
Sudan	14 (4.7)	34.0 (2.0)	
Jordan	9 (3.0)	35.0 (6.0)	
India	78 (26.0)	25.0 (15.0)	
Pakistan	25 (8.3)	23.0 (18.0)	
Sri Lanka	11 (3.7)	16.0 (24.0)	
Nepal	5 (1.7)	22.0 (8.0)	
Bangladesh	31 (10.3)	17.0 (8.0)	
Philippines	12 (4.0)	33.0 (13.0)	
Iran	2 (0.7)	34.5 (-)	
Others	26 (8.7)	36.0 (3.0)	
Education level			
Primary school	7 (2.3)	16.0 (6.0)	≤0.001^§^
Middle school	27 (9.0)	17.0 (8.0)	
High school	84 (28.0)	19.0 (9.0)	
College/diploma	21 (7.0)	31.0 (15.0)	
University	144 (48.0)	34.0 (4.0)	
Post-graduate	16 (5.3)	35.0 (3.0)	
Languages spoken^†^			
Arabic			
Yes	161 (53.7)	33.0 (13.0)	0.001^‡^
No	139 (46.3)	23.0 (17.0)	
English			
Yes	266 (88.7)	31.0 (16.0)	0.014^‡^
No	34 (11.3)	26.0 (15.0)	
Hindi			
Yes	122 (40.7)	22.0 (16.0)	≤0.001^‡^
No	178 (59.3)	33.0 (11.0)	
Urdu			
Yes	103 (34.3)	22.0 (16.0)	≤0.001^‡^
No	197 (65.7)	33.0 (13.0)	
Tamil			
Yes	3 (1.0)	27.0 (-)	0.788^‡^
No	297 (99.0)	31.0 (16.0)	
Tagalog			
Yes	14 (4.7)	30.0 (17.0)	0.806^‡^
No	286 (95.3)	31.0 (16.0)	
Malayalam			
Yes	39 (13.0)	24.0 (14.0)	0.035^‡^
No	261 (87.0)	31.0 (16.0)	
Other			
Yes	60 (20.0)	22.0 (18.0)	≤0.001^‡^
No	240 (80.0)	32.0 (14.0)	
Occupation			
Unemployed	21 (7.0)	20.0 (19.0)	≤0.001^§^
Management	27 (9.0)	34.0 (5.0)	
Finance/accounting	20 (6.7)	34.0 (3.0)	
Medical/healthcare	12 (4.0)	36.0 (0.0)	
Driving	20 (6.7)	17.50 (6.0)	
Retail salesperson	13 (4.3)	20.0 (12.0)	
Retired	17 (5.7)	29.0 (13.0)	
Administration	24 (8.0)	33.0 (7.0)	
Engineering	29 (9.7)	34.0 (4.0)	
Teaching	6 (2.0)	35.5 (3.0)	
Labor	39 (13.0)	18.0 (9.0)	
Cashier	2 (0.7)	24.5 (-)	
Secretary	2 (0.7)	17.5 (-)	
Others	68 (22.7)	31.0 (13.0)	
Health information source^†^			
Physician			
Yes	280 (93.3)	31.0 (17.0)	0.472^‡^
No	20 (6.7)	30.5 (12.0)	
Pharmacist			
Yes	234 (78.0)	32.0 (14.0)	0.008^‡^
No	66 (22.0)	25.5 (17.0)	
Physiotherapist			
Yes	29 (9.7)	35.0 (4.0)	≤0.001^‡^
No	271 (90.3)	25.5 (16.0)	
Nurse			
Yes	200 (66.7)	32.0 (14.0)	0.004^‡^
No	100 (33.3)	27.0 (16.0)	
Dietician			
Yes	16 (5.3)	31.5 (13.0)	0.902^‡^
No	284 (94.7)	31.0 (16.0)	
Person in charge of medications (at home)			
Self	298 (99.3)	31.0 (16.0)	0.652^§^
Spouse/partner	2 (0.7)	26.0 (-)	
Diagnosis			
HF only	32 (10.7)	33.0 (14.0)	0.791^§^
ACS only	237 (79.0)	31.0 (16.0)	
HF and ACS	31 (10.3)	29.0 (13.0)	
NYHA classification**			
I	13 (21.0)	35.0 (4.0)	0.043^§^
II	31 (50.0)	31.0 (12.0)	
III	14 (22.6)	24.0 (16.0)	
IV	4 (6.5)	20.5 (17.0)	
ACS type**			
STEMI	90 (39.6)	28.0 (16.0)	0.127^§^
NSTEMI	108 (47.6)	31.0 (16.0)	
UA	29 (12.8)	33.0 (15.0)	
Comorbidities^†^			
Diabetes			
Yes	145 (48.3)	31.0 (15.0)	0.749^‡^
No	155 (51.7)	31.0 (17.0)	
Hypertension			
Yes	199 (66.3)	32.0 (15.0)	0.067^‡^
No	101 (33.7)	27.0 (16.0)	
Dyslipidemia			
Yes	123 (41.0)	32.0 (14.0)	0.011^‡^
No	177 (59.0)	28.0 (17.0)	
Renal dysfunction			
Yes	18 (6.0)	30.0 (17.0)	0.766^‡^
No	282 (94.0)	31.0 (16.0)	
Liver dysfunction			
Yes	2 (0.7)	29.0 (-)	0.538^‡^
No	298 (99.3)	31.0 (16.0)	
Atrial fibrillation			
Yes	18 (6.0)	34.0 (11.0)	0.130^‡^
No	282 (94.0)	31.0 (16.0)	
Other			
Yes	78 (26.0)	33.0 (16.0)	0.415^‡^
No	222 (74.0)	30.5 (16.0)	
Smoking status			
Never	169 (56.3)	31.0 (17.0)	0.495^§^
Former	73 (24.3)	29.0 (13.0)	
Current	58 (19.3)	29.0 (15.0)	
S-TOFHLA category			
Adequate (23–36)	192 (64.0)	34.0 (13.0)	≤0.001^‡^
Inadequate or marginal (0–22)	108 (36.0)	17.0 (7.0)	
BHLS category			
Adequate (10–12)	139 (46.3)	35.0 (4.0)	≤0.001^‡^
Inadequate or marginal (0–9)	161 (53.7)	21.0 (13.0)	

**Notes**: *S-TOFHLA scores range from 0 to 36. **Missing values. ^†^Items are not mutually exclusive. ^‡^P-values were calculated using Mann Whitney-*U* test. ^§^P-values were calculated using Kruskal Wallis test.

**Abbreviations**: ACS, Acute Coronary Syndrome; BHLS, Brief Health Literacy Screen; HF, Heart Failure; NSTEMI, Non-St-Elevation Myocardial Infarction; NYHA, New York Heart Association; S-TOFHLA, short version of the Test of Functional Health Literacy in Adults; STEMI, ST-Elevation Myocardial Infarction; UA, Unstable Angina.

Patients with NYHA Class I and Class II had higher health literacy scores than those with NYHA Class III and Class IV. Further, patients with dyslipidemia had significantly higher HL score than those with no such comorbidity. However, no differences were found for most other co-morbidities. As expected, participants with adequate health literacy had significantly higher health literacy scores compared to those with inadequate or marginal health literacy. In addition, for the purpose of triangulation, the same comparisons (demographic characteristics, clinical characteristics, and health literacy levels) were repeated using 3-item BHLS health literacy categorization and the findings were similar.

Among all patients’ characteristics, there was a significant positive correlation between the number of comorbidities and S-TOFHLA (r=0.138, p=0.017). All other demographic and clinical characteristics did not show significant correlation with either S-TOFHLA or 3-item BHLS scores.

## Discussions

### Key Findings

Patients’ health literacy is a critical determinant of patients’ active participation in their healthcare decision and disease management. In particular, adequate health literacy is essential for adherence and better health outcomes among patients with CVDs. Our study determined the prevalence of health literacy and identified the associations between demographic characteristics that may be used to identify Middle Eastern patient’s with CVD at risk of having low or marginal health literacy. The S-TOFHLA was used to assess the functional health literacy of the patients, whereas the 3-item BHLS helped to assess the overall confidence of patients in health-related tasks. These two different health literacy assessment instruments were utilized for triangulation purposes.

The present study has established that 36% of patients with ACS and/or HF had inadequate or marginal health literacy based on S-TOFHLA, while more than 50% had inadequate or marginal health literacy based on 3-item BHLS. These findings demonstrate an alarming low health literacy level among patients with CVDs in Qatar. These results are in line with a number of previous studies conducted to assess the prevalence of low health literacy.^14–16^,^37^ For instance, in the first extensive national adult literacy assessment conducted in United States (US), it was found that 36% of adults had either below basic or basic health literacy.^37^ In addition, only 12% of the adult population was proficient in health literacy.^37^ However, the levels of health literacy specifically among patients with ACS and/or HF reported in the literature varied according to the region and the setting where the study was conducted. The prevalence of low health literacy among patients with ACS in the US was 34%.^15^ However, another study conducted in a similar setting indicated a prevalence rate of 44% among patients with ACS.^16^ Moreover, a systematic review reported that the prevalence of low health literacy among HF patients varied greatly from 17.5% to 97%, with an average of 39% of study participants having low health literacy.^14^ Therefore, our study results regarding the prevalence of low health literacy among patients with ACS or HF reaffirm the results reported by other studies conducted elsewhere.

There were some differences between patients who had adequate health literacy and those who had inadequate or marginal health literacy in terms of some demographic characteristics, including, educational level, spoken languages and socioeconomic status. These characteristics are widely recognized as factors associated with health literacy in the literature. This study found that 78% of the patients with university education had adequate health literacy, while less than 10% had inadequate or marginal health literacy. The median (IQR) S-TOFHLA score for patients with high school education or less ranged from 16 (6) to 19 (9) as compared to 34 (4) to 35 (3) for patients with undergraduate or postgraduate university education. Final interpretation of our study results and published literature highlight that the education background, patient’s knowledge, and past experiences, are important factors that influence patients’ capacity to look for and comprehend health information, specifically in identifying trusted sources of health information.^38^,^39^ However, other studies have also shown that attainment of high levels of education does not guarantee having high levels of health literacy.^38^,^40–45^

In concurrence with previous studies, this study established that patients who are not proficient in the main language of the country where they receive healthcare, in this case Arabic, tend to have lower levels of health literacy since the language barrier is a barrier for effective communication.^40^,^42^,^44–51^ The median (IQR) S-TOFHLA scores differed significantly according to the language spoken by patients. In addition, 63% of patients who could read and write in Arabic (the official language in Qatar) had adequate health literacy, whereas 37% had inadequate or marginal health literacy. Conversely, of the sampled patients who could speak Hindi, 29% had adequate health literacy compared to 62% who had inadequate or marginal health literacy. Communication between patients and healthcare providers is an integral component of health literacy.^48^ Patients of different nationalities would most likely face difficulty in understanding and communicating with healthcare providers due to language barriers. Previous studies have identified patients’ spoken language as one of the main factors affecting communication and health literacy.^44^

Patients’ income level, which could be related to their occupation, was also found to be a contributing factor to health literacy in previous studies.^38^,^42^,^43^,^45^,^48^,^51^ While all of the participants who worked as drivers had inadequate or marginal health literacy, only about 7% of the participants who had managerial positions had inadequate or marginal health literacy. At the social level, lack of family support has been identified as a barrier for health literacy.^38^,^42–44^,^48^,^50^,^51^ The present study revealed some potential differences in health literacy levels based on marital status. The median (IQR) S-TOFHLA score was 19 (16) among single patients as compared to 31 (15) among married patients. A systematic review of the perspectives of healthcare providers and patients on health literacy found that the lack of family support is among the perceived barriers.^42^ Jordan et al also concluded that having a good support system, including family support, was associated with higher levels of health literacy.^38^

These findings suggest the need for identifying the prevalence of limited health literacy and recognize the characteristics of patients with limited health literacy. This would be helpful to identify this segment of patients who may need targeted interventions the most. The findings indicate the need for effective strategies, tools, and interventions to assist healthcare professionals in improving health literacy among patients with ACS and HF, which can potentially improve health outcomes in this population.^17^ For effective self-management, ACS or HF patients’ ability to read, assess, comprehend medical information, make informed decisions, and access appropriate healthcare has to be improved.^10^,^11^ Patient health literacy is an important element of effective health information sharing as well as self-management of chronic diseases.^12^ Lack of skills in these areas caused by limited health literacy can undoubtedly restrict many ACS and HF patients from being involved in effective self-care management of their conditions. This is because patients with limited health literacy tend to let their healthcare providers make important decisions regarding their health without their input.^52^ One of Qatar’s current national strategy targets is patient empowerment through knowledge and health literacy as well as active involvement of community in raising health awareness, promotion of healthy behaviors, and creation of a culture of public participation.^53^

### Implications for Future Research, Policy and Practice

This research regarding the prevalence of limited health literacy will play an important role in the development of policies, strategies, and interventions designed to improve health literacy among patients with CVDs. As Qatar’s healthcare system has become focused in adopting prevention and self-management strategies, more effective solutions are required. Ultimately, the result may lead to improvements in knowledge, health literacy skills, self-management skills, and health outcomes.

## Limitations

This study has some limitations, the majority of which are inherent to its cross-sectional survey design. Although it was planned to include patients who speak common languages in Qatar including Arabic, English, Hindi, Urdu, Tamil, Tagalog, and Malayalam, the study included only patients who could speak Arabic and/or English. This is because the validity of the instruments in languages beside English and Arabic could not be established. Therefore, participants whose native language was neither Arabic nor English or were unable to speak these languages were under-represented, subjecting the study to selection bias. Consequently, the findings may not be generalized to all patients with CVDs in Qatar or the Arab world. Although generic questionnaires allow cross-condition comparison and comparison with healthy individuals, one of their limitations is that they may be less responsive to detect and quantify subtle changes related to a specific disease.^25^ On the other hand, disease-specific instruments focus on specific aspects of a particular disease and are more sensitive to measure small changes that can be important to clinicians and patients.^25^ It is worthwhile to note that the study sample may not be representative of the CVD population in the Gulf Cooperation Council (GCC) or the Middle East region. The reason is that there are some demographic differences between the countries. In addition, the findings were prone to social desirability bias as the measurement of the level of health literacy was through interviewer-administered technique. Finally, the health literacy assessment tools used (S-TOFHLA and BHLS), although widely used, are not disease-specific; however, the patients had multiple comorbidities making it impossible to have the participant fill out several disease-specific health literacy instruments.

## Conclusion

The health literacy level observed among patients with CVDs, particularly ACS and HF, in this study was low. This indicates that many CVD patients would struggle to understand various health-related information and instructions needed to manage their health conditions. Healthcare providers in cardiology settings should take extra care when educating patients, taking into consideration patients with limited health literacy. In addition, appropriate strategies and interventions should be developed and implemented to address health literacy issues. These could include utilizing patient-centered communication, improving educational materials, training healthcare providers, and employing a multilingual staff. There is a need to design studies that assess tools and interventions for the improvement of health communication and health outcomes among patients with CVD and low health literacy; these studies should investigate and evaluate the impact of improving literacy on health outcomes of patients with CVDs.
